# Enantioselective Determination of Upadacitinib in Pharmaceutical Formulations Using Reversed‐Phase Chiral Liquid Chromatography

**DOI:** 10.1002/jssc.70531

**Published:** 2026-09-09

**Authors:** Sakine Atila Karaca, Sona Aliyeva, Duygu Yeniceli

**Affiliations:** ^1^ Department of Analytical Chemistry Faculty of Pharmacy Anadolu University Eskisehir Turkey; ^2^ Graduate Education Institute Anadolu University Eskisehir Turkey

**Keywords:** chiral separation, design of experiments, enantiomeric purity, upadacitinib, validation

## Abstract

Stereochemical impurities can significantly alter a drug's pharmacological profile and compromise patient safety even in small levels. Therefore, the development of selective chiral analytical methods is critical for quality control assurance. In this study, a novel chiral HPLC method was developed for the enantiomeric separation and quantification of upadacitinib, a selective JAK‐1 inhibitor used in the treatment of rheumatoid arthritis, psoriatic arthritis, ulcerative colitis, and Crohn's disease. The Box–Behnken design, a design of experiments approach, was applied to evaluate the effects of mobile phase pH, acetonitrile content, and column temperature on resolution and peak width responses. Based on the design of experiment results, the chromatographic conditions were optimized to achieve optimal separation performance. Chiral separation was achieved under reversed‐phase conditions using a Chiralcel OJ–RH column (4.6 × 150 mm, 5 µm) at 40°C. The mobile phase consisted of 20 mM phosphate buffer (pH 6) and acetonitrile (84:16, v/v), delivered isocratically at a flow rate of 0.7 mL/min, with detection performed at 340 nm. Baseline enantiomeric separation was achieved with a resolution value of 2.51. The method was validated according to ICH Q2(R2) guidelines in terms of specificity, linearity, accuracy, precision, and robustness. Good linearity was obtained over the concentration range of 0.1–12.8 µg/mL for upadacitinib and its 3*R*,4*S* enantiomer. The validated method was successfully applied to the quantification of upadacitinib in a commercial tablet formulation. Its environmental impact and practicality were evaluated using various tools. This study presents the first validated reversed‐phase chiral HPLC method reported for the enantiomeric analysis of upadacitinib.

## Graphical abstract

A reversed‐phase chiral HPLC method was developed for the enantioselective separation and quantification of upadacitinib using a Box‐Behnken design. The validated method was successfully applied to the quantification of upadacitinib in a commercial tablet formulation, and its environmental impact and practicality were evaluated using various assessment tools.

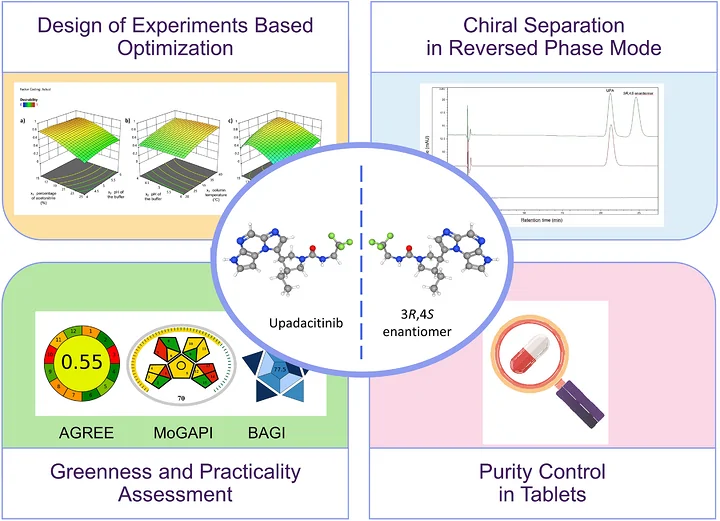

## 1 Introduction

Janus kinase (JAK) inhibitors have been widely used for the treatment of immune dysfunction diseases such as rheumatoid arthritis, psoriatic arthritis, ulcerative colitis and Crohn's disease. Among these, upadacitinib (UPA) is a selective JAK‐1 inhibitor that has more than 60‐fold selectivity compared to JAK‐2 and more than 100‐fold selectivity compared to JAK‐3 in cellular assays; this selectivity reduces side effects associated with the inhibition of JAK‐2 and JAK‐3. In 2019 the US Food and Drug Administration (FDA) approved UPA for the treatment of rheumatoid arthritis. It was also approved for psoriatic arthritis in 2021, ulcerative colitis in 2022 and Crohn's disease in 2023 [1, 2, 3, 4].

According to a recent review on enantioselective synthetic strategies for chiral kinase inhibitors, no studies have yet evaluated the effects of UPA stereoisomers on JAK1 or other isoforms. Furthermore, the original synthesis patent of UPA reports only selected assay results for JAK3 inhibitors, without including UPA or its antipode. However, among the selected examples, the JAK3 inhibitory activity range is available for an analogue of upadacitinib (AA.1.77 according to the patent numbering) and its enantiomer (AA.1.76). While the compound AA.1.77 presented an IC50 value of <0.1 µm, the enantiomer was inactive (IC50 >1 µm) indicating the critical role of the stereochemistry for this class of compounds [5, 6].

Relatively few analytical procedures for UPA quantification have been published in the literature, including HPLC [7, 8], LC–MS/MS [7, 9, 10, 11, 12, 13], electrochemical techniques [14, 15, 16] and other methods [17]. In addition to all these achiral methods, a chiral method has also been reported [18], in which the separation of UPA and its enantiomer was achieved under normal‐phase (NP) conditions employing a ChiralPak IG column.

In this study, chiral separation of UPA was developed using a ChiralCel OJ‐RH column under reversed‐phase (RP) conditions with a mobile phase including a low percentage of acetonitrile (16%). The RP mode has several advantages over the NP mode including better solubility of polar compounds, simpler sample preparation from biological samples and the use of less expensive and more environmentally friendly solvents [19]. The applicability of RP methods for LC‐MS applications has been another reason to favor them over the past ten years [20, 21]. Generally, the chiral separations in the RP mode are characterized by a lower consumption of organic solvents and an equal or higher potential for the successful separation of enantiomers [19, 20, 21, 22].

The Box–Behnken design (BBD) was implemented to systematically explore the variables affecting the enantioseparation. This design was chosen to improve time efficiency, reduce the overall workload and avoid experiments under extreme conditions [23]. Mathematical models were constructed to describe both the linear and quadratic relationships among critical parameters. Response surface methodology was subsequently applied to evaluate the individual and interactive effects of these variables and to identify the optimal chromatographic conditions.

The environmental impact of the proposed method was evaluated according to the Green Analytical Chemistry (GAC) principles [24]. Method greenness was assessed using the Analytical GREEnness (AGREE) and the modified green analytical procedure index (MoGAPI) metrics, which consider parameters such as solvent consumption, chemical hazards, and analysis time [25, 26]. The blue applicability grade index (BAGI) was additionally applied to examine whether environmental performance was maintained alongside analytical suitability for routine use [27].

This study presents the first validated RP chiral HPLC method reported for UPA. The method was developed through a design of experiments‐based optimization strategy and was intended for routine quality control applications while maintaining a favorable environmental profile.

## 2 Materials and Methods

### 2.1 Chemicals and Materials

The reference standards of UPA and the 3*R*,4*S* enantiomer were obtained from BLD Pharm (Shanghai, China). The HPLC‐grade methanol and acetonitrile were purchased from Sigma–Aldrich (Germany) and Carlo Erba (France), respectively, while the ultrapure water was produced using a Merck Milli‐Q purification system.

The potassium phosphate dibasic (K_2_HPO_4_) was supplied by VWR Chemicals (Belgium) while the sodium hydroxide was obtained from Merck (Germany). The phosphoric acid (85.0–88.0%) was purchased from Sigma–Aldrich (Switzerland) while the hydrochloric acid (36.5%–38%) and hydrogen peroxide (30%) were from Sigma–Aldrich (Germany).

### 2.2 Instrumentation and Chromatographic Conditions

An analytical balance (Mettler Toledo XP205, Switzerland) and a centrifuge (Eppendorf 5810 R, Germany) were used for the sample preparation. The chromatographic analyses were performed on an Agilent 1290 Infinity Binary HPLC system (Agilent Technologies, USA), consisting of a degasser, binary pump, autosampler, column compartment, and a diode array detector.

Chiral separation was achieved using a Chiralcel OJ‐RH column (4.6 × 150 mm, 5 µm, Daicel), packed with a polysaccharide‐based chiral stationary phase. The mobile phase consisted of a phosphate buffer (20 mM, pH 6) and acetonitrile (84:16, v/v) delivered isocratically at a flow rate of 0.7 mL/min. The column temperature was set at 40°C, the injection volume was 10 µL and the detection wavelength was 340 nm.

### 2.3 The Preparation of the Standard and Sample Solutions

The stock solutions of the UPA and the 3*R*,4*S* enantiomer (1 mg/mL) were prepared individually in methanol and stored at ‐20°C. An intermediate mixed solution (100 µg/mL of each analyte) was then prepared using a methanol:water (50:50, v/v) mixture as a diluent. The calibration standards (0.10–12.80 µg/mL) and the quality control samples (0.25, 2.50, and 10.00 µg/mL), were obtained by serial dilution of this intermediate solution with the same diluent.

For the sample preparation, a commercial UPA tablet formulation (Rinvoq, labeled 15 mg/tablet) was finely powdered. An amount equivalent to 1 mg of UPA was dissolved in 1 mL methanol and it was centrifuged at 4000 rpm for 10 min. The supernatant was collected, filtered through a 0.22 µm cellulose acetate–cellulose nitrate syringe filter, and appropriate dilutions were then made to obtain a final concentration of 2.5 µg/mL for analysis.

### 2.4 Method Validation Study

Prior to the validation studies, system suitability was verified to confirm consistent chromatographic performance. This was carried out through three replicate injections of the standard solution (2.5 µg/mL), and parameters such as the retention factor (*k*), tailing factor (*T*), resolution (*R_S_*), and relative standard deviation (%RSD) of retention time and peak area were evaluated.

The method validation was performed according to the ICH Q2(R2) guideline, by evaluating specificity, linearity, precision, accuracy, stability, and robustness [28]. The chromatographic peak areas were used as the analytical response throughout the validation studies.

The specificity was confirmed by the absence of interfering peaks in blank samples and further supported by peak purity analysis based on spectral data. In addition, forced degradation studies were performed to evaluate the stability‐indicating capability of the method.

The linearity was assessed using eight calibration levels between 0.1 and 12.8 µg/mL, each analyzed in triplicate. The calibration curves were created by plotting peak area against concentration. The quantitation limit (QL) was directly verified at the lower range limit of 0.1 µg/mL based on accuracy and precision measurements, in accordance with ICH Q2(R2) [28].

The accuracy and precision (intraday and inter‐day) of the developed method were evaluated using quality control samples at three concentration levels. In addition, the accuracy and precision of the method were specifically evaluated for the enantiomeric impurity at 0.1 µg/mL in the presence of UPA at 100 µg/mL [29, 30, 31]. The accuracy was reported as the percentage recovery of the added analyte, while the precision was expressed as the %RSD. All of the analyses were performed in six replicates to ensure the reliability of the results.

The robustness was evaluated using a standard solution containing 2.5 µg/mL UPA. The impact of the minor, deliberate variations in critical parameters, such as the buffer pH and concentration, flow rate, and column temperature was assessed by monitoring recovery values.

Stability studies for the UPA solutions were also performed at three concentration levels (0.25, 2.5, 10 µg/mL). For short‐term stability, the solutions were maintained at room temperature for 24 h. For long‐term stability, they were stored at −20°C for a period of 2 weeks. In addition, the effect of the repeated freeze‐thawing was evaluated after three freeze–thaw cycles.

### 2.5 Forced Degradation Studies

Forced degradation studies were performed to verify the selectivity of the method and its ability to resolve the analyte in the presence of potential degradation products. A standard solution (2.5 µg/mL) was subjected to acid hydrolysis (0.1 M HCl), base hydrolysis (0.1 M NaOH) at both room temperature and 70°C, while oxidative stress (1% v/v H_2_O_2_) was performed at room temperature. All the stressed samples were monitored at 3, 6, and 24‐h intervals to evaluate the degradation profile. The chromatograms were examined to confirm that the analyte peaks were well‐resolved from any degradation products. In addition, peak purity was assessed to further support this evaluation.

### 2.6 Greenness and Practicality Assessment

The greenness and practical applicability of the method were evaluated using AGREE, MoGAPI, and BAGI metrics [25, 26, 27]. All the assessments were performed through their corresponding web‐based tools [32, 33, 34].

AGREE and MoGAPI were used to quantify the overall environmental impact of the method, including solvent usage, sample preparation requirements, and instrumental conditions. BAGI was applied to evaluate the practical applicability of the method in routine laboratory use. Together, these metrics allowed both environmental and practical aspects of the method to be considered.

### 2.7 Software

Statistical evaluation and data processing were performed using Microsoft Excel and Design‐Expert 13 software packages.

## 3 Results and Discussion

### 3.1 Preliminary Screening and Parameter Selection

To identify the most influential factors for the enantiomeric separation of UPA, preliminary screening studies were first conducted using a one‐factor‐at‐a‐time methodology. Initially, the suitability of the stationary phase was evaluated using two different polysaccharide‐based columns: ChiralPak IC (4.6 × 250 mm, 5 µm), containing cellulose tris(3,5‐dichlorophenylcarbamate) immobilized on silica, and ChiralCel OJ‐RH (4.6 × 150 mm, 5 µm), featuring cellulose tris(4‐methylbenzoate) coated on silica gel. Since superior enantioresolution was observed in the ChiralCel OJ‐RH column, further method development was carried out using this column.

Interestingly, in our recent study on talazoparib using the same two columns, better enantioseparation was achieved on ChiralPak IC, rather than on ChiralCel OJ‑RH [35]. This observation highlights the analyte‐dependent nature of chiral recognition and the complementary behavior of different cellulose‐based stationary phases [36, 37]. Consistent with this, the present results indicate that structurally related compounds may exhibit different selectivity profiles depending on the chiral selector.

During the preliminary optimization studies, acetonitrile and methanol were compared as organic modifiers in the mobile phase. While methanol resulted in poor enantiomeric separation, acetonitrile provided significantly better resolution. Furthermore, the effect of the mobile phase pH was examined using acidic, neutral, and basic buffers. Under acidic conditions, low retention prevented effective separation, whereas a considerable improvement in resolution was achieved under near‐neutral conditions (pH 6.0).

The column temperature was also an important factor affecting enantioseparation. However, the operational limitations of the stationary phase were a critical constraint. According to the manufacturer's recommendations, the chiral column should not be operated above 25°C at pH values higher than 7. For this reason, subsequent experiments were performed within a restricted pH range and under controlled temperature conditions to avoid potential damage to the column.

The detection wavelength was set at 340 nm based on the UV‐Vis absorption spectra of the analytes. This wavelength provided adequate sensitivity and an improved signal‐to‐noise ratio for the simultaneous determination of UPA and its enantiomer.

### 3.2 The Experimental Design‐Based Method Optimization

Following the preliminary screening, a BBD was employed to optimize the separation conditions. The percentage of acetonitrile, buffer pH, and column temperature were selected as independent variables since the initial screening experiments showed that these parameters had the greatest influence on chromatographic behavior. Peak width and resolution were monitored as response variables to evaluate separation performance, particularly with respect to enantioseparation. The experimental levels assigned to each factor are summarized in Table S1.

Based on the BBD matrix (Table S2), fifteen experiments were carried out in randomized order. The experiments under all conditions were performed in triplicate using a 2.5 µg/mL mixture of UPA and its enantiomer. The experimental data were statistically evaluated by way of Design‐Expert 13, yielding quadratic response surface models (Equation 1) for each investigated response.

(1) $$\begin{array}{ccc} Y & = & b_{0}+b_{1}x_{2}+b_{2}x_{2}+b_{3}x_{3}+b_{2}x_{2}+b_{11}x_{1}^{2}+b_{22}x_{2}^{2}\\ & & +\;b_{33}x_{3}^{2}+b_{12}x_{1}x_{2}+b_{13}x_{1}x_{3}+b_{23}x_{2}x_{3} \end{array}$$

In this equation, *Y* corresponds the response, *b* to the regression coefficients, and *x*_1_, *x*_2_, and *x*_3_ to the experimental factors.

As presented in Table 1, all the models were statistically significant (*p* < 0.05). The high coefficient of determination (*R* ^2^) values indicated the robustness and predictive reliability of the developed models. The ANOVA results suggested that the chromatographic responses were primarily governed by the main effects of the investigated factors. Of these factors, the acetonitrile content had the strongest impact on both the peak width and the resolution. Higher acetonitrile ratios reduced the peak width but also led to lower enantioresolution. Buffer pH mainly affected the resolution, whereas the elevated column temperatures generally improved separation behavior.

**TABLE 1:** Regression coefficients and statistical significance for the Box–Behnken design.

Coefficients	R_S_	W_A_	W_B_
b_0_	1.08* [0.96, 1.20]	0.288* [0.127, 0.450]	0.340* [0.119, 0.561]
b_1_	−0.685* [−0.758, −0.612]	−0.437* [−0.536, −0.338]	−0.601* [−0.736, −0.465]
b_2_	0.148* [0.075, 0.222]	0.028 [−0.071, 0.127]	0.065 [−0.070, 0.200]
b_3_	0.344* [0.271, 0.418]	−0.110* [−0.209, −0.011]	−0.157* [−0.292, −0.022]
b_12_	−0.020 [−0.124, 0.084]	−0.038 [−0.177, 0.102]	−0.114 [−0.305, 0.078]
b_13_	0.085 [−0.019, 0.189]	0.161* [0.021, 0.301]	0.215* [0.024, 0.406]
b_23_	−0.026 [−0.130, 0.078]	0.001 [−0.139, 0.141]	0.004 [−0.188, 0.195]
b_11_	0.044 [−0.064, 0.152]	0.273* [0.128, 0.419]	0.411* [0.212, 0.610]
b_22_	−0.122* [−0.230, −0.014]	−0.067 [−0.212, 0.079]	−0.075 [−0.274, 0.124]
b_33_	−0.049 [−0.157, 0.059]	0.062 [−0.084, 0.208]	0.069 [−0.130, 0.268]
Model's *p* value	< 0.0001	0.0023	0.0020
R^2^	0.9935	0.9719	0.9732
Adjusted R^2^	0.9818	0.9214	0.9250

*Note*: Values in brackets represent the 95% confidence intervals (CI).

Abbreviations: *R_S_*, resolution; *W_A_*, width of UPA; *W_B_*, width of 3*R*,4*S* enantiomer.

* A statistically significant model term at a 95% confidence level.

Several interaction and quadratic terms were also found to be significant, indicating that the responses were influenced by combined and nonlinear effects in addition to the individual contribution of each factor. In particular, the interaction between the acetonitrile content and the column temperature affected peak width noticeably.

**FIGURE 1:**
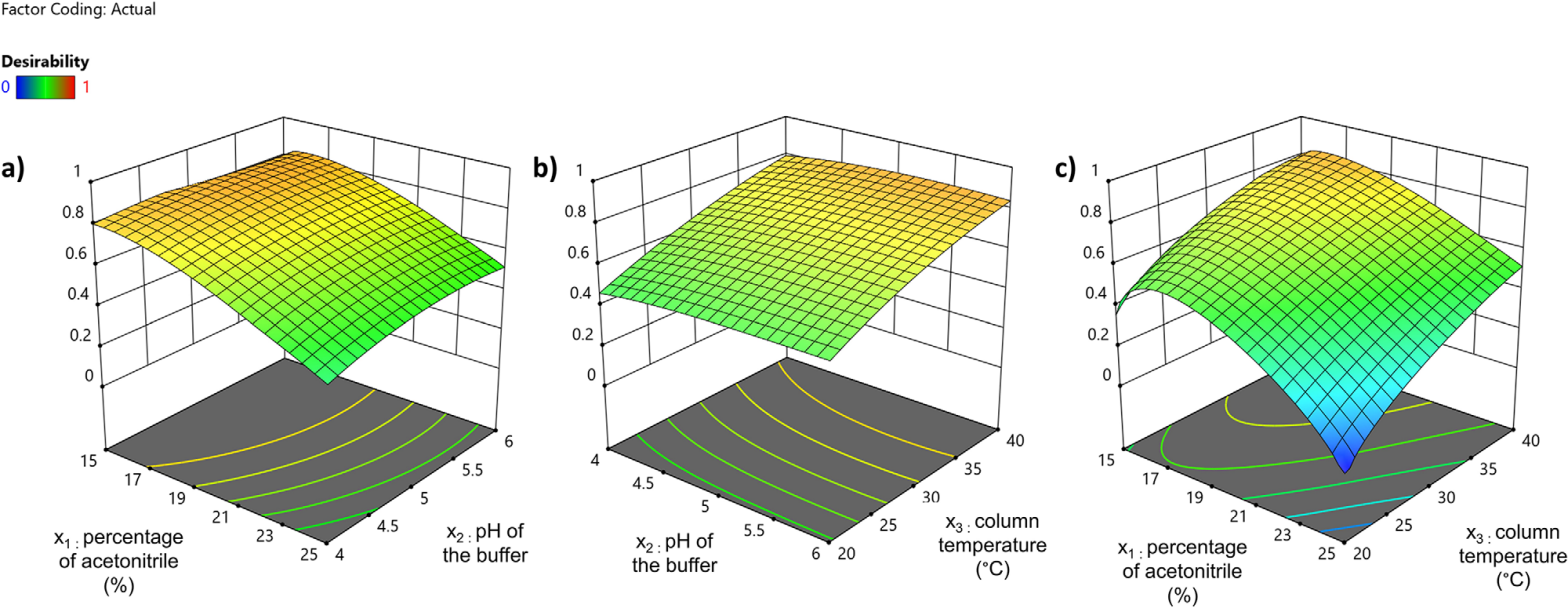
Surface graphics in experimental design. Graphics showing the interaction between (a) percentage of acetonitrile (x_1_) and pH of the buffer (x_2_); (b) pH of the buffer (x_2_) and column temperature (x_3_); and (c) percentage of acetonitrile (x_1_) and column temperature (x_3_).

The optimal conditions were determined by maximizing the overall desirability function within the Design‐Expert software. Specific objectives were set for each response, and the highest‐scoring conditions were selected. Figure 1 presents the three‐dimensional response surface plots of the desirability function. These plots depict the interaction effects between the pairs of variables, while the third factor is kept constant.

Based on the optimization, the optimal conditions were identified as 16% acetonitrile, 20 mM phosphate buffer at pH 6, and a column temperature of 40°C. The experimental results showed good agreement with the model predictions, with a deviation of approximately 5%. Subsequent flow‐rate optimization revealed that 0.7 mL/min provided improved enantioseparation. The final chromatographic conditions therefore consisted of 16% acetonitrile and 20 mM phosphate buffer (pH 6) as the mobile phase, a flow rate of 0.7 mL/min, a column temperature of 40°C, an injection volume of 10 µL, and UV detection at 340 nm.

### 3.3 Method Validation

The system suitability parameters of the optimized method are summarized in Table 2. All observed values were within the acceptance criteria [38], confirming the consistent performance of the system. The high resolution between UPA and its enantiomer demonstrates that the method is suitable for its intended chiral separation purpose.

**TABLE 2:** System suitability parameters (*n* = 3).

Parameters	Observed value		Requested value
	UPA	3*R*,4*S* enantiomer	
Retention time (min)	21.05	24.51	—
Retention factor (*k*)	8.85	10.38	2‐10
Tailing factor (*T*)	1.10	1.09	≤ 1.2
Resolution (*R_S_*)	—	2.51	>2
RSD (%) of retention time	0.62	0.62	< 1
RSD (%) of peak area	0.64	0.64	< 1

Abbreviation: RSD, relative standard deviation.

**FIGURE 2:**
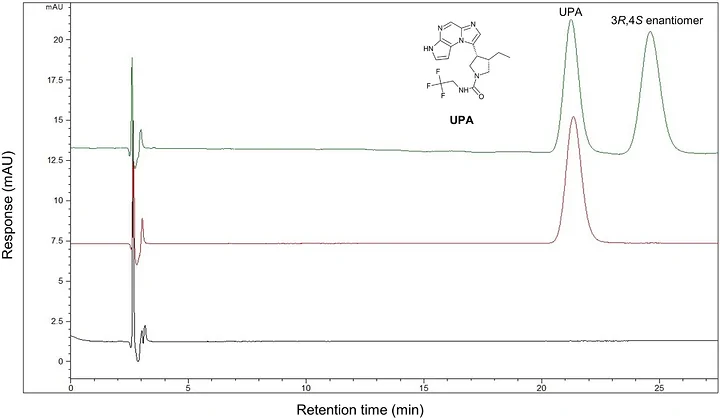
Chromatograms of (a) UPA and *3R,4S* enantiomer mixture (2.5 µg/mL); (b) tablet (containing 2.5 µg/mL UPA); and (c) blank analyses under optimal conditions.

The developed analytical method was validated in accordance with the ICH Q2(R2) guidelines [28]. The method specificity was confirmed by the absence of any interfering peaks at the retention times of the UPA and 3*R*,4*S* enantiomer in blank chromatograms (Figure 2). Peak purity analysis, based on photodiode array spectra, indicated that each peak represented a single, pure component. As discussed in detail in Section 3.4, forced degradation studies further supported the method selectivity, as no co‐eluting peaks were observed under the applied stress conditions within the detection limits of the method. These findings indicate that the method is suitable for the selective quantification of UPA and its enantiomer in the presence of potential degradation products.

The method showed good linearity for UPA and its enantiomer over a concentration range of 0.1–12.8 µg/mL. The calibration curves were constructed using the least‐squares regression method, and the corresponding statistical parameters are presented in Table 3. In all cases, *R* ^2^ values were higher than 0.999, indicating a strong correlation between concentration and response. QL value was established at 0.1 µg/mL for both UPA and its enantiomer based on accuracy and precision measurements at this concentration (Table 3).

**TABLE 3:** Linearity data and statistical parameters of the calibration curve.

Parameters	UPA	3*R*,4*S* enantiomer
Range (µg/mL, *n = *8)	0.1–12.8	0.1–12.8
Slope (mean ± SD)	153.99 ± 0.19	172.35 ± 0.20
Intercept (mean ± SD)	1.47 ± 0.98	−1.92 ± 1.06
Coefficient of determination (R^2^)	0.9999	0.9999
QL (µg/mL)	0.10	0.10
Recovery at QL (%, *n = *6)	102.31	100.54
Precision at QL (%RSD, *n = *6)	1.24	1.76

Abbreviation: SD, standard deviation.

**FIGURE 3:**
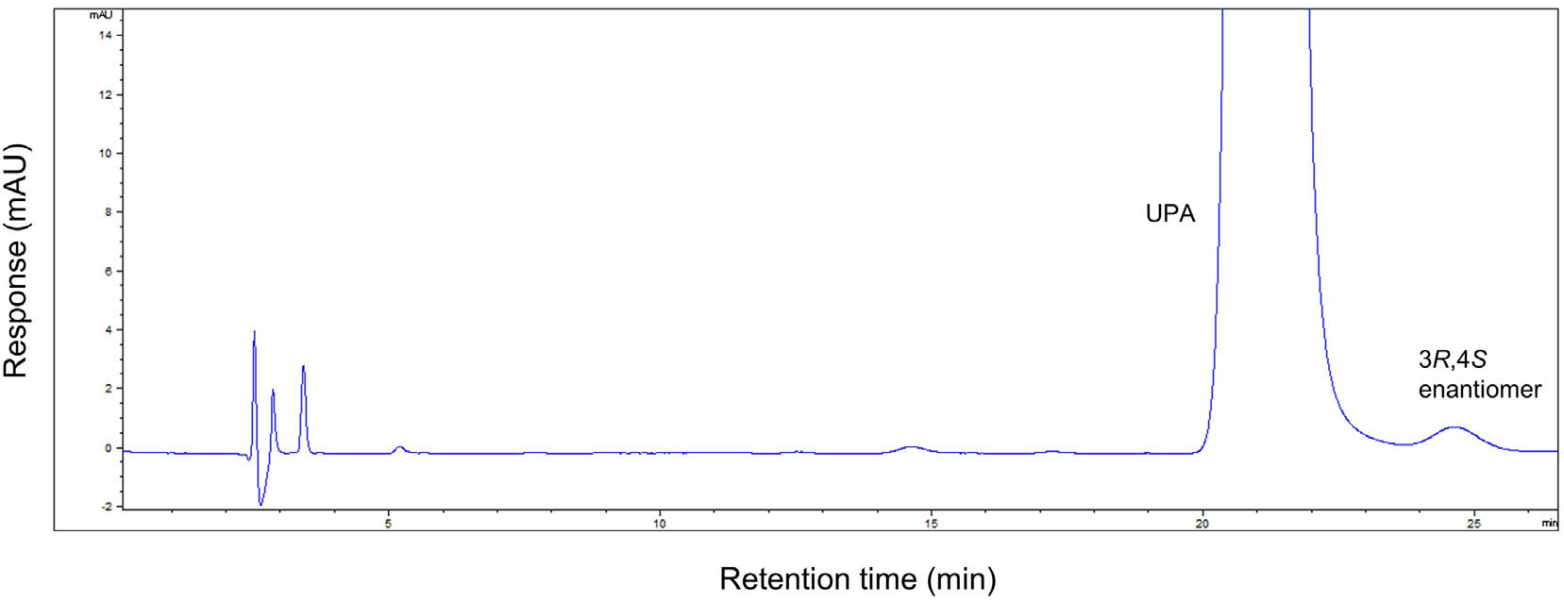
Chromatogram of UPA (100 µg/mL) and *3R,4S* enantiomer (0.1 µg/mL) mixture.

The accuracy and precision were evaluated at three quality control levels. Recovery values ​​ranging from 97% to 105% confirmed the accuracy of the method, while RSD values ​​below 5% confirmed its precision (Table 4). In addition, the accuracy and precision of the 3*R*,4*S* enantiomer at 0.1 µg/mL were evaluated in the presence of UPA at 100 µg/mL in both a standard solution and a pharmaceutical tablet solution. Mean recoveries of 98.78% and 98.07% were obtained for the standard and tablet solutions, with %RSD values of 1.20% and 1.45% (*n = *6), respectively. These results demonstrate the accurate and precise quantification of the enantiomeric impurity at the 0.1% level in the presence of UPA at the 100% level. The chromatogram obtained from the spiked standard solution is presented in Figure 3.

**TABLE 4:** Accuracy and precision data.

Analyte	Added Concentration (µg/mL)	Intraday (*n = *6)		Interday (*n = *18)	
		Recovery (%)	RSD (%)	Recovery (%)	RSD (%)
UPA	0.25	99.21	3.32	98.53	2.96
	2.5	102.26	0.31	99.86	1.89
	10	99.63	0.50	97.27	1.81
3*R*,4*S* enantiomer	0.25	100.17	2.99	103.71	4.08
	2.5	103.18	0.42	105.04	1.48
	10	101.69	0.39	103.07	1.25

Abbreviation: RSD, relative standard deviation.

The robustness of the method was evaluated by introducing small deliberate changes in the critical chromatographic parameters, as summarized in Table S3. Under all these conditions, recovery values ranged from 95.03% to105.3%. These findings indicate that the developed method is not significantly affected by minor operational variations that may occur during routine analysis.

Stability studies showed that the UPA standard solutions remained stable under different storage conditions, including short‐term room temperature exposure, long‐term refrigeration at ‐20°C, and multiple freeze–thaw cycles. In all the storage conditions, recovery variations were below 6% (Table S4).

### 3.4 The Forced Degradation Studies

The stability‐indicating capability of the method was evaluated by subjecting the standard UPA solution to various stress conditions. No significant degradation was observed during the initial 3‐h exposure, with recovery values remaining within 95%–102%. However, the 24‐h experiments provided a more detailed profile of the analyte's stability (Table 5).

**TABLE 5:** Results of the forced degradation studies (*n = *3).

Stress conditions		UPA			
		Recovery (%)	RSD (%)	Purity factor	Purity threshold
Room temperature	Base hydrolysis (0.1 M NaOH, 24 h)	88.01	0.24	999.968	999.771
	Acid hydrolysis (0.1 M HCl, 24 h)	101.03	0.69	999.958	999.864
	Oxidation (1% H_2_O_2,_ 24 h)	99.03	0.14	999.984	999.849
70°C	Base hydrolysis (0.1 M NaOH, 24 h)	86.66	0.06	999.974	999.679
	Acid hydrolysis (0.1 M HCl, 24 h)	96.82	0.49	999.971	999.719

The UPA showed relatively stable behavior under acidic and oxidative conditions, whereas notable degradation occurred under alkaline stress. After 24 h of exposure to 0.1 M NaOH, recoveries decreased to approximately 88% at room temperature and 86% at 70°C. Despite this decrease, no additional chromatographic peaks attributable to degradation products were detected within the method's detection limits.

Peak purity analysis also confirmed the spectral homogeneity of the UPA peak, with purity values remaining above the acceptance threshold throughout the experiments. These results confirm that the method effectively separates UPA from its degradation products and is suitable as a stability‐indicating analytical procedure.

### 3.5 Tablet Analysis

The developed method was applied to the analysis of UPA in Rinvoq tablets to assess its enantiomeric purity. The tablet sample was analyzed in triplicate at a medium concentration (2.5 µg/mL), yielding a recovery rate of 96.76% and an RSD of 0.027%. Figure 2 shows a comparison of the mixture of UPA and its enantiomer, the tablet sample, and the blank analyses.

### 3.6 The Greenness and Practicality Assessment of the Proposed Method

The environmental sustainability of the developed chiral separation method was evaluated using widely accepted GAC assessment tools, namely AGREE, MoGAPI, and BAGI.

Acetonitrile was used as the organic modifier in the mobile phase in the present study. The dipolar aprotic and water‐miscible solvent acetonitrile is a commonly used eluent in RP chromatography because of its advantages including low viscosity and low UV absorption (>190 nm) [39, 40, 41, 42]. Moreover, acetonitrile demonstrated moderate performance, considering both Physical and Health Hazards categories (moderate score 7 in Physical Hazards category and low score 3 in Health Hazards category) according to a comprehensive solvent selection guide named GreenSOL. It is less hazardous than many chlorinated or aromatic hydrocarbons, but it still presents risks, particularly due to its flammability and its potential for inhalation exposure at high concentrations. Also, GreenSOL reported that, acetonitrile achieves low scores (1–3) in the waste phase assessment especially in recycling and incineration categories [41].

The AGREE score was calculated as 0.55 (on a scale of 0–1), indicating a moderately green analytical performance (Table 6). This value primarily reflects the use of acetonitrile as the organic modifier and the offline nature of the analytical workflow. Although used in relatively low proportions, the inherent environmental and toxicological profile of acetonitrile influenced the overall assessment.

**TABLE 6:** Comparison of the proposed method with the previously reported chiral NP‐HPLC method.

Parameters		Reported Method [18]		Proposed Method	
Analytical parameters	Technique	NP‐HPLC		RP‐HPLC	
	Column	ChiralPak IG column (150 × 4.6 mm, 5 µm)		Chiralcel OJ‐RH column (150 × 4.6 mm, 5 µm)	
	Stationary Phase	Amylose tris(3‐chloro‐5‐methylphenylcarbamate)		Cellulose tris(4‐methylbenzoate) coated on silica‐gel	
	Mobile Phase	n‐hexane/ethanol (70:30, v/v)		phosphate buffer (20 mM, pH 6): acetonitrile (84:16, v/v)	
	Flow Rate	1.8 mL/min		0.7 mL/min	
	Run Time	15 min		30 min	
	Detection	UV detection at 230 nm		UV detection at 340 nm	
	Column temperature	40°C		40°C	
		UPA (mg/mL)	3*R*, 4*S* enantiomer (µg/mL)	UPA (µg/mL)	3*R*, 4*S* enantiomer (µg/mL)
Validation	Linearity Range	0.33–2.0	0.34–2.0	0.1–12.8	0.1–12.8
	Quantification limits	0.33	0.34	0.10	0.10
Application	Sample	Tablets		Tablets	
	Sample preparation	Extraction in 75 mL ethanol		Extraction in 1 mL methanol	
Greenness	AGREE^*^	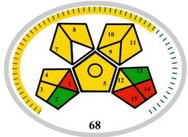		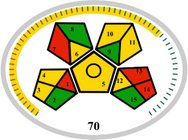	
	MoGAPI	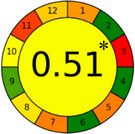		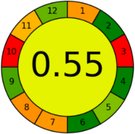	
Practicability	BAGI	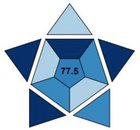		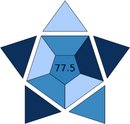	

* AGREE score of reference method was calculated by the authors based on the reported experimental conditions.

The MoGAPI evaluation yielded a score of 70 (out of a maximum 100), indicating good overall sustainability. As shown in the MoGAPI pictogram (Table 6), positive contributions were observed in areas such as simple sample preparation, limited reagent consumption, and effective waste handling. Lower scores were associated with the use of organic solvents (acetonitrile and methanol), offline operation, potential vapor emission and waste volume.

Beyond the environmental considerations, the BAGI score was determined to be 77.5 (out of a maximum 100) as illustrated in Table 6. This result indicates that the developed procedure is practical for routine use while maintaining reasonable analytical performance.

Overall, considering the intrinsic limitations of the chiral chromatographic separations, such as the use of organic modifiers and offline procedures, the proposed method achieves a balanced compromise between analytical performance and environmental impact. The combined AGREE, MoGAPI, and BAGI evaluations indicate that the method is both practically applicable and reasonably sustainable. In light of previous reports on the use of more environmentally friendly solvent alternatives, such as ethanol, to acetonitrile in RP‐chromatography, the potential of these solvents to enhance the analytical performance of the current method warrants further investigation in future studies [39, 40, 41, 42].

## 4 Conclusion

Given that even trace amounts of stereochemical impurities can significantly alter a drug's pharmacological profile and compromise patient safety, the development of selective chiral analytical methods is crucial for rigorous quality control. Supporting this, pronounced stereochemical and pharmacological differences have been reported for structurally related analogues of UPA [5, 6]. For this purpose, an RP‐HPLC method was developed and validated for the chiral separation of UPA and its enantiomer in this study. Considering the advantages of RP chiral separations discussed above, the proposed method offers practical benefits in terms of solvent compatibility, simplified sample preparation, and broader applicability, including potential coupling with LC–MS systems.

A literature survey indicates that a NP chiral HPLC method has previously been reported for UPA using a ChiralPak IG column [18]. A comparative evaluation of the proposed RP‐HPLC method and the reported NP‐HPLC method is presented in Table 6, including analytical conditions, method performance parameters, and greenness performance. The environmental impact and practical applicability comparison were based on the reported MoGAPI and BAGI evaluations. In addition, to ensure a more objective evaluation, the AGREE score for the NP‐HPLC method was independently calculated from the experimental conditions reported in the literature. As shown in Table 6, although the NP method provides shorter analysis time, the n‐hexane in the mobile phase raises environmental and safety concerns [36]. This difference is also reflected in the lower AGREE score of the NP method compared to the proposed RP approach. In contrast, the proposed RP method reduces the use of hazardous solvents and decreases overall solvent consumption through a lower flow rate and simplified sample preparation.

To the best of our knowledge, this study presents the first validated RP chiral HPLC method for the enantioselective determination of UPA in pharmaceutical formulations. The method was successfully validated in terms of selectivity, linearity, accuracy, precision, and robustness. The developed method exhibited high sensitivity, achieving QL of 0.1 µg/mL for both UPA and its enantiomer. This concentration was lower than that previously reported for 3*R*,4*S* enantiomer using the NP‐HPLC method (0.34 µg/mL). Also, 0.1% level of the enantiomeric impurity could be accurately and precisely quantified in the presence of the active ingredient at the 100% level. This characteristic enables more reliable determination at low concentration levels and supports its suitability for routine quality control applications.

Overall, the proposed RP‐HPLC method offers a reliable and sensitive approach for routine quality control and enantiomeric purity assessment of UPA, with improved environmental performance compared to conventional methods.

## Author Contributions

**Sakine Atila Karaca**: writing – original draft, methodology, software, data curation, supervision, visualization, formal analysis. **Sona Aliyeva**: writing – original draft, validation, investigation, formal analysis, software, methodology, data curation, visualization, conceptualization. **Duygu Yeniceli**: conceptualization, methodology, supervision, funding acquisition, resources, project administration, writing – review and editing.

## Conflicts of Interest

The authors declare no conflicts of interest.

## Supporting information




**Supporting File**: jssc70531‐sup‐0001‐SuppMat.docx.

## Data Availability

The data that support the findings of this study are available from the corresponding author upon reasonable request.
