# Supplementary material for: Enantioselective Determination of Upadacitinib in Pharmaceutical Formulations Using Reversed‐Phase Chiral Liquid Chromatography
Source: J Sep Sci. 2026 Sep 9;49(9):e70531. doi: 10.1002/jssc.70531 (PMC13555205; doi:10.1002/jssc.70531)
Supplement: Supplementary file 1 [file JSSC-49-e70531-s001.docx]

**SUPLEMENTARY MATERIAL**

**Enantioselective Determination of Upadacitinib in Pharmaceutical Formulations Using Reversed-Phase Chiral Liquid Chromatography**

Sakine Atila Karaca*^[a]^, Sona Aliyeva^[b]^, Duygu Yeniceli ^[a]^

**^[a]^** Department of Analytical Chemistry, Faculty of Pharmacy, Anadolu University, Eskisehir, Turkey.

**^[b]^** Graduate Education Institute, Anadolu University, Eskisehir, Turkey.

**^*^**Corresponding author, e-mail: sakineatila@anadolu.edu.tr

**Table S1.** Factors and levels for BBD.

| Factors | Levels | | |
| --- | --- | --- | --- |
|  | -1 | 0 | +1 |
| x₁ - percentage of acetonitrile (%) | 15 | 20 | 25 |
| x_2_ - pH of the buffer | 4 | 5 | 6 |
| x_3_ - column temperature (°C) | 20 | 30 | 40 |

**Table S2**. Experimental plan and responses for BBD.

| Standart | Factors | | | Responses | | |  |
| --- | --- | --- | --- | --- | --- | --- | --- |
|  | x₁ | x_2_ | x_3_ | *R_S_* | *W_A_* | *W_B_* | |
| 1 | 15 | 4 | 30 | 1.51 | 0.56 | 0.69 | |
| 2 | 25 | 4 | 30 | 0.18 | 0.11 | 0.14 | |
| 3 | 15 | 6 | 30 | 1.87 | 0.96 | 1.44 | |
| 4 | 25 | 6 | 30 | 0.46 | 0.13 | 0.15 | |
| 5 | 15 | 5 | 20 | 1.56 | 1.45 | 1.94 | |
| 6 | 25 | 5 | 20 | 0.02 | 0.30 | 0.30 | |
| 7 | 15 | 5 | 40 | 1.97 | 0.68 | 0.74 | |
| 8 | 25 | 5 | 40 | 0.76 | 0.12 | 0.13 | |
| 9 | 20 | 4 | 20 | 0.35 | 0.30 | 0.36 | |
| 10 | 20 | 6 | 20 | 0.67 | 0.35 | 0.42 | |
| 11 | 20 | 4 | 40 | 1.20 | 0.22 | 0.24 | |
| 12 | 20 | 6 | 40 | 1.42 | 0.24 | 0.27 | |
| 13 | 20 | 5 | 30 | 1.14 | 0.28 | 0.33 | |
| 14 | 20 | 5 | 30 | 1.08 | 0.29 | 0.34 | |
| 15 | 20 | 5 | 30 | 1.03 | 0.30 | 0.36 | |

*R_S_* : resolution, *W_A_* : width of UPA, *W_B_* : width of 3*R*,4*S* enantiomer.

**Table S3**. Robustness results for the optimized method (2.5 μg/mL, n=3).

| Parameter | Altered condition | UPA | 3*R*,4*S* enantiomer |
| --- | --- | --- | --- |
|  |  | Recovery (%) | Recovery (%) |
| pH of the buffer | 5.9 | 97.21 | 103.85 |
|  | 6.1 | 96.79 | 103.45 |
| Buffer Concentration | 18 | 97.06 | 104.92 |
|  | 22 | 97.49 | 104.54 |
| Flow rate (mL/min) | 0.68 | 100.18 | 105.39 |
|  | 0.72 | 95.30 | 101.83 |
| Column temperature (°C) | 39 | 97.09 | 102.04 |
|  | 41 | 97.85 | 103.64 |
| Detector wavelength | 338 | 95.03 | 100.41 |
|  | 342 | 97.72 | 103.64 |

**Table S4**. Stability data (n=3).

| Analyte | Concentration  (μg/mL) | Short-term stability  (24 h, room temperature) | | Freeze-thaw stability  (3 cycles) | | Long-term stability  (2 weeks, -20ºC) | |  |
| --- | --- | --- | --- | --- | --- | --- | --- | --- |
|  |  | Recovery (%) | RSD (%) | Recovery (%) | RSD (%) | Recovery (%) | RSD (%) | |
| UPA | 0.25 | 97.65 | 1.56 | 98.43 | 1.80 | 97.26 | 1.71 | |
|  | 2.5 | 96.04 | 0.54 | 98.13 | 0.13 | 101.58 | 0.54 | |
|  | 10 | 93.45 | 0.06 | 94.92 | 0.02 | 101.38 | 0.39 | |
| 3*R*,4*S* enantiomer | 0.25 | 99.53 | 1.89 | 106.18 | 1.56 | 100.17 | 1.47 | |
|  | 2.5 | 99.98 | 0.24 | 106.51 | 0.38 | 102.60 | 0.08 | |
|  | 10 | 96.86 | 0.04 | 103.38 | 0.05 | 101.99 | 0.46 | |
